# Dual roles of circadian clock proteins in development and adult homeostasis

**DOI:** 10.1038/s44323-025-00061-1

**Published:** 2026-01-09

**Authors:** Matthew Choi, Stacy Liang, Juan R. Alvarez-Dominguez

**Affiliations:** 1 Chronobiology and Sleep Institute, Institute for Diabetes, Obesity and Metabolism, and Institute for Regenerative Medicine, University of Pennsylvania Perelman School of Medicine, Philadelphia, PA USA; 2 Department of Cell and Developmental Biology, University of Pennsylvania Perelman School of Medicine, Philadelphia, PA USA

**Keywords:** Cell biology, Developmental biology

## Abstract

Circadian clocks coordinate physiology with daily cycles. Loss of circadian components disrupts bodily functions, but the phenotypes differ when disruption occurs during development or adulthood. Here, we compare effects of manipulating mammalian clock proteins across life stages, reviewing roles of BMAL1 and REV-ERBα/β in embryogenesis—skeletal formation, metabolic programming, cardiovascular development—and adulthood—tissue integrity, metabolic adaptation, neuroprotection. We further discuss mechanisms for dual involvement, and implications for stage-specific therapies.

## Introduction

Like Earth’s rotation, our bodies follow an autonomous 24-hour cycle, known as the circadian rhythm^1^. This system influences critical physiological processes, including metabolism^2^, sleep-wake cycles^3^, and blood pressure^4^. Its autonomy was first observed in plants, which continued to open and close their leaves even without light and dark cues from sunlight^5^. In mammals, scientists have since uncovered the specific proteins that program autonomous circadian rhythms through a transcription-translation feedback loop^6^.

**Fig. 1:**
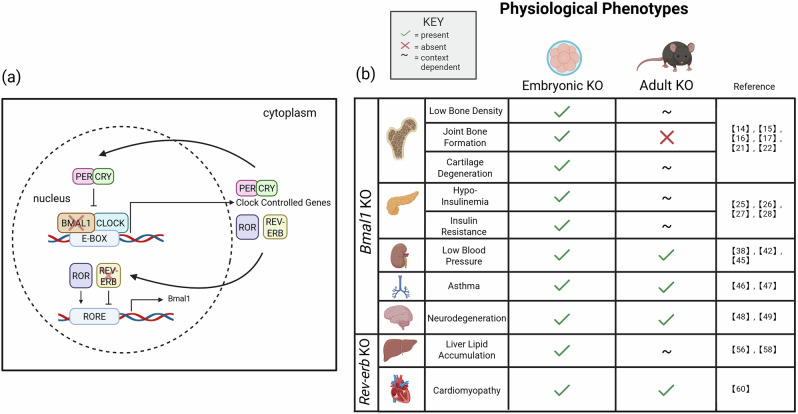
Molecular feedback loop of the circadian clock and comparative physiological phenotypes in Bmal1 and Rev-erb knockout models. **a** Molecular feedback loop of the circadian clock, involving BMAL1, CLOCK, PER, CRY, ROR, and REV-ERB proteins. **b** Comparison of physiological phenotypes observed in embryonic vs. adult *Bmal1* or *Rev-erb* knockout (KO) models. Check mark (✓), (X), and tilde (~) symbols indicate the presence, absence, or context-dependency of a phenotype, respectively. Created with BioRender.com.

The core activating proteins, BMAL1 and CLOCK, form heterodimers that bind to DNA E-box elements of target genes, including their own, to induce their transcription. These targets include PER and CRY, which form a complex that represses CLOCK and BMAL1 activity, creating a transcription feedback loop (Fig. 1a) that lasts about 24 hours^7^. This 24-hour clock is modulated by additional transcription factors, including RORα/β and REV-ERBα/β proteins, that activate or repress BMAL1 expression. Such interlocking layers of regulation allow fine-tuning the circadian clock to adjust time-of-day variation in specific bodily functions, like our daily metabolic, body temperature, digestion, bone growth, hormonal, heart rate, and blood pressure rhythms^8^.

The 24-hour periodicity of circadian rhythms is genetically encoded and remains stable despite physiological changes. Circadian rhythm amplitude and phase, on the other hand, adapt to environmental cues—such as light, temperature, and feeding^9^—that synchronize clocks across various tissues. These cues help align circadian rhythms by adjusting the peak time of cellular processes (phase synchronization) and the strength of oscillations (amplitude control) without altering the fundamental 24-hour cycle.

Inactivating clock genes disrupts circadian rhythmicity. However, the physiological effects depend on when inactivation occurs—whether during development or in adulthood. During development, the circadian clock plays crucial roles in tissue formation, differentiation, and metabolic programming. Developmental clock disruption can thus lead to long-term structural and functional deficits. Alternatively, it may trigger compensatory adaptive mechanisms that mask functional deficits. Adult-life clock disruption bypasses developmental defects, by contrast, allowing investigation of the clock’s roles in regulating physiological homeostasis. Beyond circadian control, clock proteins may also play non-circadian roles, both during development and in adult homeostasis.

Here, we review the roles of BMAL1 and REV-ERBα/β in development and adult physiology. We focus on these proteins due to their well-understood molecular mechanisms and loss-of-function phenotypes. BMAL1 and REV-ERBs regulate each other and are broadly expressed across many tissues. Yet their expression level, rhythmicity, and functions can vary by tissue and developmental stage. BMAL1 is lowly expressed in early embryogenesis, but increases significantly during late fetal and early postnatal stages, along with the onset of circadian gene expression in peripheral tissues^7^. In contrast, REV-ERBα and β are expressed earlier during development and become particularly prominent in the developing brain and liver. The presence of BMAL1 and REV-ERBs before circadian rhythms are detected suggests roles during development, including in metabolic programming and cellular differentiation.

We review the functions of BMAL1 and REV-ERBα/β in bone and cartilage homeostasis, glucose metabolism, blood pressure regulation, airway function, neuroprotection, and cardiovascular homeostasis (Fig. 1b). We chose these tissues and processes since they offer strong mechanistic evidence linking BMAL1 and REV-ERBs disruption to disease. While BMAL1 influences many other processes, these areas offer robust models and translational potential. In addition, REV-ERBs have been extensively studied in the context of liver metabolism and myocardial dysfunction, underscoring their relevance to cardiometabolic health. We compare findings from embryonic vs. adult and global vs. tissue-specific mouse knockout models. This lets us distinguish and mechanistically dissect developmental from homeostatic functions, as well as potential compensatory mechanisms. Finally, we discuss the therapeutic potential of targeting circadian pathways in a life stage-specific manner to treat metabolic disorders.

### BMAL1

#### BMAL1 in Bone and Cartilage

The skeletal system comprises bone and cartilage made of mesenchymal stem cell-derived osteocytes and chondrocytes, respectively. The balance between bone/cartilage resorption and formation is a tightly regulated, timed process^10^. Xu et al. showed that bone resorption in fully developed osteoclasts is higher in the dark (active) phase of the day, and is compromised in embryonic osteoclast-specific *Bmal1* KO mice^11^. Bone growth is also under clock control, since embryonic *Bmal1* KO in mouse chondrocytes and fibroblast-like synoviocytes leads to impaired joint development, including abnormal cartilage formation and altered cellular composition within the synovium^12^. Disrupted circadian rhythms, in turn, can alter cartilage breakdown or bone remodeling patterns, leading to diseases such as osteoarthritis or osteoporosis. In humans, chronic circadian disruption due to rotating work schedules (shift work) compromises bone health, fueling bone fracture risk^13,14^.

**Fig. 2:**
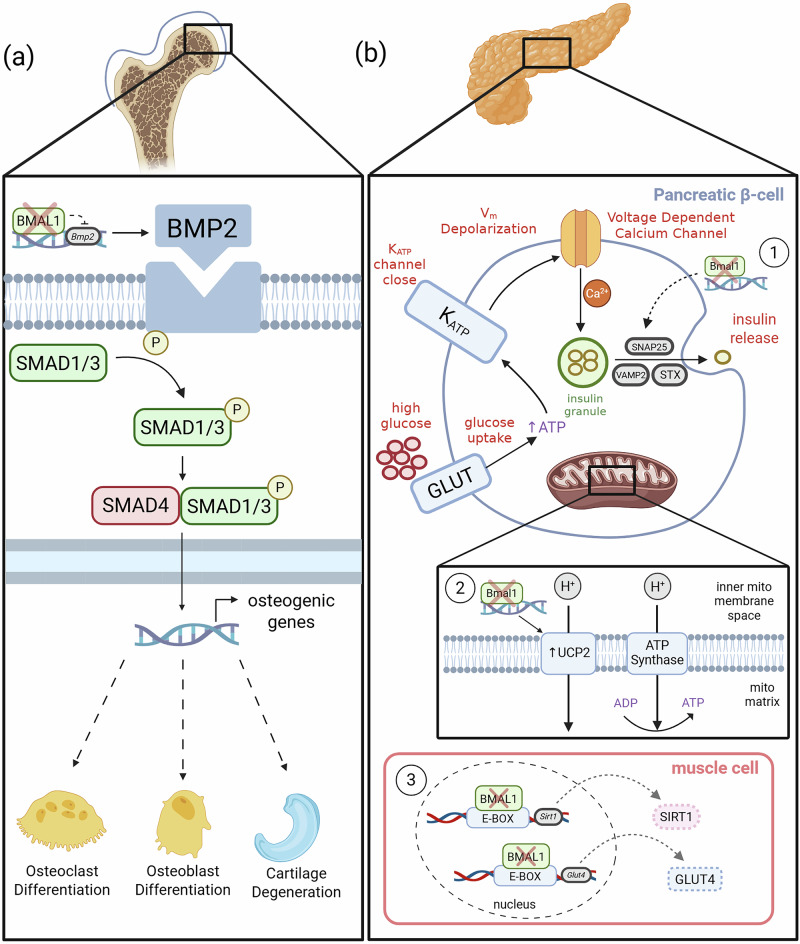
Proposed BMAL1-dependent regulation of the BMP pathway and mechanisms underlying metabolic dysfunction in Bmal1-knockout mice. **a** Proposed model for BMAL1-dependent regulation of the BMP pathway. Under normal conditions, BMAL1 binds the *Bmp2* promoter and suppresses BMP2 ligand expression^23^. Loss of BMAL1 upregulates BMP2, leading to enhanced phosphorylation of downstream SMAD1/3 effectors^17,23^. Together, these changes induce osteogenic genes, impacting differentiation in osteoblasts and osteoclasts^15–17,23^ and inciting cartilage degeneration^18–20^ due to ectopic ossification in joints. **b** Proposed mechanisms contributing to diabetic phenotypes in BMAL1-KO mice: 1) BMAL1 loss disrupts the rhythmic transcriptional regulation of late exocytic machinery in pancreatic β-cells, impairing the stimulus-coupled fusion and release of insulin-containing vesicles^25,29^. 2) Upregulation of UCP2 transmembrane proteins in pancreatic β-cells lacking BMAL1, which impairs the H⁺ electrochemical gradient, disrupts mitochondrial ATP production, and thus insulin secretion^27^. 3) BMAL1 loss decreases transcription of SIRT1 and GLUT4 in skeletal muscle, reducing insulin sensitivity and impairing glucose uptake^30,31,33^. Created with BioRender.com.

BMAL1 is active throughout skeletal development and homeostasis, and regulates both bone and cartilage formation. Many skeletal phenotypes, including impaired osteogenesis, abnormal joint architecture, and ectopic ossification, arise only when BMAL1 is deleted during embryogenesis. By contrast, adult-onset deletions typically produce more site-specific or reversible changes, suggesting a role for BMAL1 in developmental programming of skeletal formation. During development, BMAL1 is essential for osteogenic differentiation, ensuring balanced bone formation (ossification) and preventing premature joint calcium deposition (calcification). In adulthood, BMAL1 maintains bone homeostasis by modulating circadian bone remodeling rhythms, preventing excessive resorption, and regulating BMP/TGFβ signaling to inhibit abnormal ossification in tendons and ligaments (Fig. 2a).

BMAL1 is essential for early skeletal development due to its roles in regulating mesenchymal stem cell differentiation, BMP/TGFβ signaling, and metalloproteinase activity. Lack of BMAL1 during development disrupts normal bone formation and joint integrity, leading to skeletal abnormalities. For example, Samsa et al.’s global embryonic *Bmal1* KO mice exhibit reduced mass and length of both trabecular and cortical bone^15^. The low bone mass phenotype of *Bmal1⁻/⁻* mice is absent at birth, but is detectable by 8 weeks (young adulthood in mice), and worsens with age, showing severe cortical/trabecular deficits and reduced osteoblast/osteocyte activity by 7–9 months. This likely stems from the reduced ability of mesenchymal stem cells to differentiate into osteoblasts, observed in vitro, leading to the low levels of active osteoblasts and osteocytes observed in vivo.

Beyond impaired bone formation, *Bmal1* deficiency also affects joint integrity. Bunger et al. reported that global embryonic *Bmal1* KO mice develop abnormal joint phenotypes, characterized by cartilage degeneration and increased ossification and calcification in the joints, which are only apparent as they age, not during development^16^. Liang et al. further showed that BMAL1 suppresses BMP/TGFβ signaling, and its absence activates this pathway, leading to increased ossification and calcification in tendons and ligaments^17^. These phenotypes were seen as early as birth. Since these effects occurred in mice housed under 12 h:12 h light-dark cycles, without assessing time-of-day variation, it is uncertain whether BMAL1’s regulation of BMP/TGFβ reflects circadian-dependent or independent roles in skeletal formation^17^. Additionally, cartilage degeneration in global *Bmal1*-deficient mice can be explained by increased metalloproteinase activity, which accelerates extracellular matrix breakdown, contributing to joint degradation^18–20^.

Tissue-specific embryonic *Bmal1* KOs match the cartilage degeneration phenotypes of global KOs. Liao et al. found that articular-cartilage embryonic *Bmal1* KO mice have reduced chondrocyte proliferation and cartilage matrix synthesis, as well as elevated cartilage apoptosis. The phenotype worsened over time, causing no cartilage changes at 2 months, mild degeneration by 6 months, and severe osteoarthritis-like damage with elevated catabolic markers by 12 months^21^. Dudek et al. found that cartilage-specific embryonic *Bmal1* ablation led to more severe, progressively worsening lesions in the articular cartilage than those seen in global embryonic *Bmal1*-KO^22^. This was attributed to desynchrony between the chondrocyte peripheral clock and the central hypothalamic pacemaker, rather than to global loss of rhythmicity. Similarly, Hand et al. found that deleting *Bmal1* in mesenchymal cells during embryogenesis caused articular cartilage cells to lose their rhythmicity and led to disruptions in joint architecture in adult mice, causing arthritis^12^.

Adult-life *Bmal1* KOs show conflicting bone phenotypes, however. Osteoblast-specific *Bmal1* ablation in adult mice still leads to BMP/TGFβ pathway activation, consistent with BMAL1 binding and repressing the *Bmp2* promoter (Fig. 2a), but only causes decreased mass in cortical bone, not trabecular bone^23^. This contrasts with observations from Samsa et al. in global embryonic *Bmal1* KOs, in which both trabecular and cortical bone show reduced mass^15^. This discrepancy may be due to compensatory pathways in the adult KO that are absent or impaired in the global embryonic KO. The higher turnover rate of trabecular bone may also explain why osteoblast numbers increase there but remain unchanged in cortical bone, which remodels more slowly^10^. Finally, since BMP/TGFβ activation is observed in both embryonic global and adult tissue-specific KOs, it is likely that BMAL1’s role in suppressing this pathway persists into adulthood but affects bone homeostasis differently depending on when it is lost. Yang et al. also found that global *Bmal1* KO in adult mice does not increase calcification of cartilage in the Achilles’ tendon, unlike global embryonic KOs^24^. This suggests that BMAL1’s role in suppressing tendon ossification is developmentally programmed rather than continuously required in adulthood.

BMAL1’s role in the skeletal system thus appears to shift over time. While embryonic deletion results in widespread skeletal defects, adult-specific KOs show more nuanced, site-dependent effects. This suggests BMAL1 enacts context-dependent inhibition of pathways regulating bone formation, such as BMP/TGFβ signaling (Fig. 2a). The diurnal regulation of bone resorption is clearly dependent on the circadian clock, whereas suppression of BMP/TGFβ signaling and prevention of ectopic ossification may reflect non-circadian roles of BMAL1. Overall, BMAL1 serves as both a developmental regulator and a homeostatic gatekeeper, integrating circadian control with skeletal health to maintain structural integrity throughout life.

#### BMAL1 in Glucose Metabolism

The circadian clock is essential for maintaining glucose homeostasis by anticipating daily energy needs. Without BMAL1, insulin secretion and glucose metabolism become impaired. Embryonic *Bmal1* KO models develop insulin deficiency, hyperglycemia, and glucose intolerance as adults, indicating that BMAL1 is critical for the function and metabolic set point programming of pancreatic β-cells. In contrast, the effects of *Bmal1* loss during adulthood are more variable or compensable: some studies report minimal impact, while others show a role in β-cell function and adaptation to metabolic stress. This suggests that BMAL1 is indispensable for metabolic programming during early development, but its necessity in adulthood depends on tissue-specific adaptations and external conditions.

Global embryonic *Bmal1* KO models show defective glucose-stimulated insulin secretion as adults^25–28^. This may be due to upregulation of UCP2, a mitochondrial uncoupling protein that decreases ATP production, in β-cells, leading to reduced insulin secretion and hypo-insulinemic diabetes^27^ (Fig. 2b). In global embryonic *Bmal1* KO mice, gluconeogenesis is completely abolished, yet corticosterone and glucagon responses to insulin-induced hypoglycemia remain unaffected^28^. Marcheva et al. found that global embryonic *Bmal1* KO and *Clock*-mutant mice develop hypo-insulinemic diabetes during adult life^25^. These mice present impaired glucose tolerance and hyperglycemia due to insufficient insulin release, which disrupts their metabolic homeostasis. Their pancreatic islets show impaired stimulus-coupled insulin secretion, attributed to disrupted transcriptional rhythms, including in exocytosis networks. Specifically, Perelis et al. found that CLOCK and BMAL1 bind pancreas-specific enhancers to impart circadian expression to genes for late secretory machinery, including vesicle transport and insulin exocytosis, in β-cells^29^ (Fig. 2b). *Bmal1* loss thus dysregulates genes controlling insulin secretion, in addition to vesicular transport and MAPK signaling pathways^25^, resulting in decreased insulin release^29^.

Tissue-specific embryonic *Bmal1* KO supports results from global embryonic KO. Pancreas-specific embryonic *Bmal1* KOs develop hyperglycemia and hypo-insulinemia as adults^12^. Additionally, they show reduced insulin release in islets stimulated by glucose or specific chemicals^25,26^. Beyond pancreatic dysfunction, BMAL1 loss disrupts insulin sensitivity in peripheral tissues. SIRT1 expression rhythms dampen in muscle-specific embryonic *Bmal1* KOs, impairing circadian insulin signaling. BMAL1 binds to E-box elements in the *Sirt1* promoter, promoting its transcription (Fig. 2b). Without BMAL1, SIRT1 levels drop, leading to reduced insulin-stimulated glucose uptake and insulin resistance in skeletal muscle^30^, manifest from early developmental stages. However, glucose tolerance remains unchanged in muscle-specific *Bmal1* KOs, suggesting that other tissues like adipose compensate to stabilize glucose uptake^31^. Liver-specific embryonic *Bmal1* KO mice also develop insulin resistance, and during adulthood show decreased ability to adapt to different nutrient conditions, such as a high-fat diet, attributed to elevated mitochondrial oxidative damage^32^.

The effects of adult-life *Bmal1* KO on glucose metabolism vary, depending on the knockout tissue and the timing of metabolic assessments. In adult muscle-specific *Bmal1* KOs, insulin resistance develops in part due to GLUT4 downregulation, which impairs glucose uptake^31,33^ (Fig. 2b). Perelis et al. found that β-cell-specific *Bmal1* KO during adulthood still causes insulin deficiency and glucose intolerance^29^. However, Yang et al. reported no significant metabolic changes following global *Bmal1* knockout in adult mice^24^. A key difference between these studies is the timing of metabolic tests—Yang et al. conducted tests 8 months after inducible adult KO, while Perelis et al. tested within 10–14 days. Evidence suggests that acute glucose intolerance and diabetic phenotypes, as observed upon *Bmal1* loss, can be compensated by age-dependent mechanisms over time^34^. Acute and long-term metabolic studies are thus needed to resolve these discrepancies.

These findings underscore BMAL1’s dual role: it is indispensable for metabolic programming during development, while in adulthood, its impact on glucose homeostasis is shaped by tissue-specific adaptations, compensatory responses, and external factors such as diet and circadian misalignment. Adult β cell-specific or global KOs can thus show from minimal to moderate metabolic changes^24,29^. BMAL1’s role in glucose metabolism thus appears to involve both developmental programming—such as regulating islet mass and secretory capacity—and lifelong circadian control, such as imparting rhythmic transcription of genes regulating glucose uptake in muscle and insulin exocytosis and signaling in β cells. Whether developmental effects arise from circadian or non-circadian roles demands further investigation.

#### BMAL1 in Blood Pressure

Blood pressure (BP) shows distinct time-of-day variation. In healthy individuals, BP has the steepest increase during a “morning surge” at the onset of waking, remains stable through the daytime with a peak in the late afternoon^35^, and then “dips” during nighttime sleeping, decreasing 10-20% relative to daytime BP^4^. Moreover, heart infarctions tend to occur earlier in the day rather than later at night^36^, highlighting the importance of studying BP in the context of circadian rhythms. BP rhythms reflect regulation by both the central nervous system and peripheral tissue clocks, especially those in the kidney, adrenal glands, nervous system, heart, and vasculature.

Global embryonic *Bmal1* KO results in a general hypotensive phenotype in adult mice^37^. This is likely due to impaired vascular smooth muscle function, as embryonic *Bmal1* KO in smooth muscle cells suppresses the maximal contractile responses of blood vessels, lowering BP^38^. Another reason could be disruption of the renin-angiotensin-aldosterone system (RAAS), which follows a circadian rhythm to regulate BP fluctuations^39^. Kidney-renin-cell-specific *Bmal1* KO during embryogenesis disrupts the rhythmic release of renin, which normally increases BP, causing a hypotensive phenotype in adult mice^40^. Thus, the renal circadian clock is essential to maintain BP homeostasis by ensuring proper RAAS responses to physiological demands. Furthermore, embryonic *Bmal1* KO in distal nephron and collecting ducts lowers baseline BP in male mice, suggesting that BP regulation relies on circadian control of sodium transport in aldosterone-sensitive regions of the kidney^41,42^.

In contrast, adult nephron-specific *Bmal1* KO mice show no changes in glomerular filtration rate, renal function, or BP regulation^43,44^. Whereas postnatal cardiomyocyte-specific *Bmal1* KO mice do exhibit enhanced BP-induced cardiac remodeling, potentially mediated by PI3K/AKT signaling^45^. This suggests that BMAL1 plays a role in adaptive cardiovascular responses, rather than baseline BP regulation, during adulthood.

In sum, BMAL1 plays distinct roles in BP regulation depending on the developmental stage. Embryonic *Bmal1* loss leads to a hypotensive phenotype in adult mice, likely due to circadian control of vascular contractility and the RAAS. This phenotype does not occur when BMAL1 is disrupted in adulthood, suggesting that while BMAL1 remains important for homeostasis, its role in BP maintenance is more nuanced and context-dependent. These findings highlight BMAL1’s dual role: during development, it is essential for establishing BP homeostasis, whereas in adulthood, it modulates physiological responses to maintain BP stability rather than acting as a primary regulator. BMAL1’s mechanisms for BP regulation thus involve both developmental programming—such as vascular and renal programming—and circadian homeostatic control, such as establishing rhythms in adaptive cardiovascular responses. Remodeling responses in cardiomyocyte-specific knockouts may represent a hybrid mechanism, with basal structural changes modulated by circadian gating under stress.

#### BMAL1 in Asthma

Circadian rhythms influence airway health and asthma development. In patients with asthma, symptoms often worsen at night or during sleep. Scheer et al. showed that this is driven by the endogenous circadian clock^46^. Using a forced desynchrony protocol to isolate circadian effects from behavioral and environmental factors, they demonstrated that airway resistance increased and pulmonary function decreased during the biological night, even in the absence of sleep or external cues, evidencing circadian control over respiratory function. Understanding circadian mechanisms in asthma pathogenesis is therefore essential. Ehlers et al. studied the effects of *Bmal1* KO in both embryonic and adult models of airway pathology^47^. They found that postnatal BMAL1 loss alone is sufficient to compromise the immune system’s ability to fight viral infections in the lung and cause asthma. This finding established BMAL1 as a key regulator of antiviral defense during both early development and adulthood. Given that immune impairment occurs even when *Bmal1* is disrupted postnatally, the study argues against developmental effects as the primary cause. Instead, it argues that BMAL1 actively regulates immune function throughout life. Further studies will be needed to delineate the specific pathways downstream of BMAL1 that modulate airway immune responses. Dissecting the temporal regulation of immune signaling, epithelial barrier integrity, and cytokine production may in turn potentiate the development of novel time-dependent therapeutic strategies for asthma.

#### BMAL1 in Neurodegeneration

BMAL1 coordinates sleep and regulates detoxification and waste clearance in the brain, which are linked to Alzheimer’s and Parkinson’s syndromes. Global embryonic *Bmal1* KO weakens the brain over time, making neurons more vulnerable to stress and aging in mice^48^. These mice show increased oxidative damage, inflammation, and loss of neural connections, especially in memory-related brain regions. Though neurons do not die immediately, they become fragile and prone to degeneration, especially under stress. Without BMAL1, these protective processes are compromised, increasing the risk of neurodegenerative disease.

Global *Bmal1* KO in adults causes rapid and direct neuron loss, particularly in dopamine-producing brain regions linked to movement^49^. Unlike embryonic loss, this kills neurons within a month, even without external triggers. The damage is not just due to circadian rhythm disruption: light-induced circadian desynchrony did not cause TH+ neuron loss, whereas neuron-restricted (Nestin-Cre) and TH-specific *Bmal1* disruption produced cell-autonomous degeneration even when behavioral rhythms were intact. BMAL1 also plays an active role in protecting neurons by supporting energy production and stress resistance, while its loss triggers gene changes linked to Parkinson’s disease^49^. This shows that BMAL1 is essential for both long-term brain health and immediate neuron survival.

Neuron-specific BMAL1 KO in adults leads to oxidative stress, synapse loss, and dopaminergic neuron degeneration, independent of disruptions in behavioral rhythms^49^. In contrast, astrocyte- or microglia-specific BMAL1 deletion does not induce dopaminergic neuron loss, though BMAL1’s role in these glial cells influences redox balance and gliosis, which are relevant to Alzheimer’s and Parkinson’s pathology.

Thus, BMAL1’s neuroprotective roles are not restricted to a developmental window. Increased oxidative stress and synapse loss occur with both developmental and adult-specific deletion, though whether early-life BMAL1 loss increases long-term vulnerability remains unknown. These effects likely reflect circadian and non-circadian functions. Oxidative stress and synapse loss occur without behavioral rhythm disruption^49^, whereas processes like glymphatic clearance and detoxification are linked to circadian cycles^7,48^. Redox cycling and proteostasis likely integrate both mechanisms.

### REV-ERBs

#### REV-ERBs in Liver Metabolism

REV-ERBs are nuclear receptors and important transcriptional repressors in the circadian clock. They play key roles in liver metabolism by preventing mistimed feeding responses and controlling energy and lipid metabolism^50^. In particular, REV-ERBβ binds and suppresses transcription of lipogenic genes, including those regulated by SREBP, a transcription factor that promotes cholesterol and fatty acid synthesis. Thus, REV-ERBβ acts as a metabolic repressor that limits the activation of the mTOR pathway and fine-tunes lipid production in the liver^51,52^. REV-ERBα similarly represses SREBP activity in hepatocytes while also playing a broader role in metabolic regulation, such as the modulation of bile acid synthesis and inhibition of TGFβ signaling, which affects brown adipose tissue differentiation^52,53^. Single REV-ERBα or REV-ERBβ KOs are viable but present developmental abnormalities when either gene is lost embryonically, or disrupted homeostasis when lost in adulthood. In contrast, double REV-ERBα and REV-ERBβ KO during embryogenesis can be lethal in global knockouts or sublethal in inducible models, depending on the tissue^54^.

Global embryonic *Rev-erbα* KO in mice disturbs SREBP activity, leading to systemic metabolic dysfunction during adulthood. This includes high lipid accumulation, causing a metabolic dysfunction-associated steatotic liver disease (MASLD) phenotype, reduced brown fat, hyperglycemia without insulin resistance, muscular atrophy, and metabolic syndrome^53–57^. REV-ERBα can also suppress TGFβ signaling. Without REV-ERBα as the repressor, increased TGFβ signaling inhibits brown adipose tissue differentiation, leading to reduced burning of excess fat^53^. Dysregulated bile accumulation is also seen, leading to oxidative stress, inflammation, and activation of the fibrosis pathway, which worsens MASLD^52^.

Unlike global embryonic REV-ERBα KOs, adult hepatocyte-specific KOs show enhanced lipid accumulation in the liver without increased body weight^58^. Similarly, adult hepatocyte-specific REV-ERBα and REV-ERBβ double KOs (hepDKO) show increased hepatic triglyceride levels^54^. This is thought to be mediated by dysregulated SREBP signaling, since loss of REV-ERBs in hepatocytes can unleash SREBP activity, causing overproduction of triglycerides^51^. Indeed, blocking SREBP signaling, via knockout of SREBP cleavage–activating protein in hepDKO mice, prevents diet-induced hepatic triglyceride accumulation^51^, restoring lipid homeostasis^59^. HepDKO mice also show lower circulating free fatty acid levels^54^, suggesting a more active uptake and utilization of fatty acids for energy through increased oxidative metabolism.

Overall, while global embryonic REV-ERBα loss causes severe systemic metabolic dysfunction, hepatocyte-specific loss of REV-ERBα or of both REV-ERBα and REV-ERBβ during adulthood causes more localized and comparatively milder effects. This indicates that REV-ERBα is critical for metabolic programming during early development. In contrast, in adulthood, the phenotype cannot be explained simply by compensation from REV-ERBβ, since deleting both paralogs in hepDKO still results in marked hepatic triglyceride accumulation^54,58^. REV-ERBs’ roles in hepatic metabolism involve both rhythmic and constitutive repression of metabolic pathways. Feeding phase-specific lipid accumulation is circadian rhythm-dependent, whereas basal suppression of SREBP activity and bile acid synthesis appears non-circadian. Mitochondrial oxidative metabolism control may involve both mechanisms, with REV-ERBs regulating both baseline respiratory activity and its alignment with circadian cues.

#### REV-ERBa in Myocardial Dysfunction

Cardiomyocyte-specific deletion of REV-ERBα and REV-ERBβ during embryogenesis is sublethal, causing postnatal heart failure and dilated cardiomyopathy^54^. The double knockout disrupts myocardial metabolic rhythms, impairing fatty acid oxidation during the light phase. This prompts a compensatory reliance on glucose metabolism in the dark (active) phase. Such a shift in fuel substrate utilization occurs whether REV-ERBs are ablated in cardiomyocytes during embryonic development or during adulthood^60^. This indicates that REV-ERBs play continuous roles in cardiac energy metabolism beyond development. Further investigation will be needed to determine if these roles depend on circadian or non-circadian mechanisms. Light-phase restricted effects on substrate utilization reflect direct circadian regulation, whereas cardiac structural remodeling in embryonic knockouts may be secondary to altered substrate utilization.

### Shared and Divergent Mechanisms Across Systems

Several unifying mechanisms emerge for BMAL1 and REV-ERB functions across skeletal, metabolic, cardiovascular, respiratory, neural, and hepatic tissues.

Coordination of mitochondrial oxidative metabolism is a recurrent thread. In pancreatic β-cells, BMAL1 loss upregulates UCP2, uncoupling oxidative phosphorylation and lowering ATP production, which limits glucose-stimulated insulin secretion^27^. In skeletal muscle, BMAL1 regulates mitochondrial oxidative metabolism via SIRT1-dependent pathways^30^. REV-ERBs likewise control mitochondrial function in hepatocytes^54^ and cardiomyocytes^60^, coordinating energy substrate utilization across light-dark phases.

Redox balance control also spans systems. BMAL1 loss in neurons and astrocytes increases oxidative stress and synapse vulnerability, even without behavioral rhythm disruption^49^. In peripheral tissues, REV-ERBs repress lipogenic and inflammatory transcriptional programs that would otherwise exacerbate oxidative imbalance, maintaining redox homeostasis in the liver^56^.

Finally, both BMAL1 and REV-ERBs influence inflammation. BMAL1 modulates cytokine production and metalloproteinase activity in joints and lungs—limiting cartilage breakdown in joints and preserving epithelial barrier integrity in the lung during viral infection^21,47^. REV-ERBα acts as a transcriptional repressor of pro-inflammatory genes in metabolic tissues and in the cardiovascular system, where its loss exacerbates maladaptive remodeling^56^.

These converging mechanisms suggest that clock proteins shape physiology not only by modulating daily fluctuations but by calibrating the baseline cellular energetic state, oxidative balance, and immune tone across organ systems.

Divergent BMAL1 and REV-ERB mechanisms stem from their opposite transcriptional roles and distinct developmental stage specialization.

BMAL1 and REV-ERBs are reciprocally linked in the circadian loop—BMAL1 drives *Rev-erb* transcription, and REV-ERBs repress *Bmal1*^6,54^. Both act through chromatin remodeling, but in opposing transcriptional modes. BMAL1 activates metabolic and structural genes via E-box–driven enhancer activity in β-cells and muscle, synchronizing transcriptional programs^25,30^. By contrast, REV-ERBs recruit NCoR/HDAC3 corepressor complexes to ROREs, restricting chromatin accessibility at metabolic and immune loci to the appropriate circadian phase^50,52,56^.

BMAL1 is critical for programming developmental processes, such as osteogenesis and β-cell maturation, with embryonic loss causing irreversible structural and metabolic defects^15,25^. Whereas REV-ERBs more often fine-tune adult homeostasis, such as limiting hepatic lipid accumulation and maladaptive cardiac remodeling^54^. As a result, BMAL1 often initiates and maintains anabolic and developmental programs, while REV-ERBs constrain these processes to the correct circadian phase and prevent overactivation.

### Gaps in the Literature

While BMAL1 and REV-ERB loss-of-function studies point to essential roles across skeletal, metabolic, cardiovascular, respiratory, neural, and hepatic systems, most of these studies cannot distinguish circadian from circadian-independent functions. Many roles—such as keeping daily rhythms in bone resorption, glucose tolerance, blood pressure, and cardiac energetics—likely reflect circadian clock functions. Others—like developmental programming of osteogenesis, β-cell maturation, and basal SREBP repression—are likely non-circadian. Yet many other roles, including in mitochondrial regulation, redox balance, inflammatory tone, and chromatin remodeling, likely reflect mixed contributions, where circadian functions gate or amplify a constitutive role.

Evidence that distinguishes between circadian, non-circadian, and mixed functions remains limited, as few studies selectively disrupt circadian expression patterns while retaining total protein levels (e.g., flattening rhythms by gene mutation or protein stability modulation). This distinction could be sharpened by comparing KO models with circadian misalignment models (e.g., shift work, chronic jet lag) in which rhythms are disrupted but protein expression is preserved. Such cross-model analyses would clarify which phenotypes arise from rhythm loss vs. constitutive protein functions in physiology and disease. Resolving these possibilities will be key to designing stage-specific interventions that target the circadian clock without perturbing non-circadian roles of clock proteins.

### Therapeutics

Drug interventions targeting the circadian clock are being explored to restore normal function in peripheral processes like insulin metabolism and lipid processing. Nobiletin, a RORα/γ agonist, can increase BMAL1 transcription in mouse and human islets and improve in vivo glucose-stimulated insulin secretion, insulin sensitivity, and lipid metabolism^61,62^. Nobiletin directly binds to and activates RORs, which then bind ROR elements within promoters of targets such as *Bmal1*, among others, to induce their transcription^61,63^. SR1078 has similarly been investigated as another RORα/γ agonist, capable of regulating gluconeogenic gene expression and glucose metabolism in mice^64^. On the other hand, REV-ERBα/β agonists, such as SR9009 and the related compound SR9011, act as metabolic repressors capable of suppressing BMAL1 transcription (among other targets), leading to reduced fat mass and improved liver health, offering potential treatments for obesity and fatty liver disease^65,66^.

The impact of RORα/γ and REV-ERBα/β agonists on insulin and lipid processing, mitochondrial function, and inflammatory pathways makes them promising candidates for harnessing the interplay between circadian rhythms and metabolic health. For example, circadian disruption has been linked to human metabolic disease^67,68^. BMAL1 is less active in pancreatic islets from type 2 diabetes, correlating with impaired insulin and glucagon secretion^62^. This suggests that altered expression of circadian controllers contributes to β-cell dysfunction in type 2 diabetes, supporting the rationale for treating these pathways pharmacologically. In mouse models, drug-induced mitigation of clock dysfunction in adults protects from diet-induced metabolic syndrome^61^. Inducible BMAL1 restoration in adult β cells of obese mice is sufficient to improve glucose-stimulated insulin secretion and glucose tolerance^61^. Similarly, REV-ERB agonists such as SR9009 have been used in adult mice to reduce fat mass and enhance liver health^65,66^. Of note, the ability of SR9009 and of SR9011 to reprogram proliferation, metabolism, and gene control in the absence of REV-ERBα and -β suggests that at least some of their effects occur via REV-ERB-independent or off-target mechanisms^69^.

Despite the efficacy of RORα/γ and REV-ERBα/β agonists in promoting metabolic health in wild-type adult mice, the effects of administering them to BMAL1 or REV-ERBα/β KO models during development have not been studied. Since nobiletin and SR1078 depend on a functional BMAL1/CLOCK complex for their effects, they are not expected or known to rescue the phenotypes of BMAL1 KO models. Yet clarifying if SR9009 or SR9011 can rescue REV-ERB KO models—whether during development or adulthood—will be critical for identifying effective treatment windows. Actionable windows may be restricted to restoring function in early-onset congenital disorders or may extend to managing dysfunction arising later in life.

Our review underscores the need to consider drugging the clock in a developmental stage-specific manner. Given distinct roles for BMAL1 and REV-ERBα/β during embryogenesis and adult homeostasis, drugging them could be harnessed to address early-life developmental disorders. Further research is needed to determine how pharmacologic modulation of circadian components at critical developmental windows may lead to new treatments for congenital metabolic conditions. Such interventions should assess drug effects with respect to their time dependence, tissue-specificity, and potential off-target actions, along with compensatory developmental changes. Understanding the interplay between circadian regulation and developmental programming will be crucial to broaden chronotherapy beyond homeostatic maintenance to developmental-stage precision interventions.

## Conclusion

The dual functions of circadian clock proteins in development and adult homeostasis highlight an intricate relationship between timekeeping mechanisms and the emergence and maintenance of a specialized physiology. Clock proteins such as BMAL1 and REV-ERBα/β regulate key developmental processes, including skeletal formation, metabolic programming, and cardiovascular development, while also contributing to homeostatic processes throughout life. Thus, the clock components may not be viewed solely as regulators of daily cycles, but as developmental determinants that establish a long-term stable physiology. The contrasting phenotypes seen when clock genes are disrupted during embryogenesis vs. adulthood suggest that circadian regulation in adulthood operates within a framework of adaptive resilience, distinct from deterministic roles in early life. This distinction has significant implications for chronotherapy and precision medicine, as targeting circadian pathways to mitigate developmental disorders or age-related diseases may require life stage-specific interventions. Ultimately, understanding the life stage specificity of clock protein functions will be necessary to identify and optimize new interventions that harness the clock’s regulatory functions to improve metabolic, cardiovascular, and neurological health.

## Data Availability

No datasets were generated or analysed during the current study.
